# Species distribution modelling for conservation of an endangered endemic orchid

**DOI:** 10.1093/aobpla/plv039

**Published:** 2015-04-21

**Authors:** Hsiao-Hsuan Wang, Carissa L. Wonkka, Michael L. Treglia, William E. Grant, Fred E. Smeins, William E. Rogers

**Affiliations:** 1 Department of Wildlife and Fisheries Sciences, Texas A&M University, College Station, TX 77843, USA; 2 Department of Ecosystem Science and Management, Texas A&M University, College Station, TX 77843, USA; 3 Biodiversity Research and Teaching Collections, Applied Biodiversity Science Program, Texas A&M University, College Station, TX 77843, USA; 4 Present address: Department of Agronomy and Horticulture, University of Nebraska, Lincoln, NE 68583, USA; 5 Present address: Department of Biological Science, University of Tulsa, Tulsa, OK 74104, USA

**Keywords:** Boosted regression trees, conservation, endangered species, Navasota ladies’ tresses, reintroduction, species distribution models

## Abstract

Concerns regarding the long-term viability of threatened and endangered plant species are increasingly warranted given the potential impacts of climate change and habitat fragmentation on unstable and isolated populations. Orchidaceae is the largest and most diverse family of flowering plants, but it is currently facing unprecedented risks of extinction. Despite substantial conservation emphasis on rare orchids, populations continue to decline. *Spiranthes parksii* (Navasota ladies' tresses) is a federally and state-listed endangered terrestrial orchid endemic to central Texas. Hence, we aimed to identify potential factors influencing the distribution of the species, quantify the relative importance of each factor and determine suitable habitat for future surveys and targeted conservation efforts. We analysed several geo-referenced variables describing climatic conditions and landscape features to identify potential factors influencing the likelihood of occurrence of *S. parksii* using boosted regression trees. Our model classified 97 % of the cells correctly with regard to species presence and absence, and indicated that probability of existence was correlated with climatic conditions and landscape features. The most influential variables were mean annual precipitation, mean elevation, mean annual minimum temperature and mean annual maximum temperature. The most likely suitable range for *S. parksii* was the eastern portions of Leon and Madison Counties, the southern portion of Brazos County, a portion of northern Grimes County and along the borders between Burleson and Washington Counties. Our model can assist in the development of an integrated conservation strategy through: (i) focussing future survey and research efforts on areas with a high likelihood of occurrence, (ii) aiding in selection of areas for conservation and restoration and (iii) framing future research questions including those necessary for predicting responses to climate change. Our model could also incorporate new information on *S. parksii* as it becomes available to improve prediction accuracy, and our methodology could be adapted to develop distribution maps for other rare species of conservation concern.

## Abstract (precis)

Navasota ladies'-tresses is an orchid native to eastern and central Texas. It was listed as endangered by the U.S. Fish and Wildlife Service in 1982 and by the State of Texas soon afterwards. Wang et al. (2015) analyzed field data collected over nine years to identify areas of critical habitat, areas into which the species could expand its range, and areas that might serve as conservation corridors. These results will provide valuable information for those interested in conservation and management of this endangered orchid as well as a framework for the development of future studies of this and other endangered plants.

## Introduction

Conservation biologists and natural resource managers are growing increasingly concerned about the manner in which climate change and accelerating habitat fragmentation may negatively affect the long-term viability of threatened and endangered plant species (Wilcove *et al.* 1998; Pitman and Jorgensen 2002; Brigham and Swartz 2003). To successfully prevent their extirpation, conservation efforts will require detailed studies of species population biology and life-history dynamics, more thorough assessments of the factors contributing to rarity, sophisticated land management and restoration strategies and the development of more robust predictive models that better identify both high-priority conservation locations as well as areas potentially suitable for plant reintroductions (e.g. Falk and Holsinger 1991; Schemske *et al.* 1994; Maschinski and Haskins 2012).

Orchidaceae is the largest and most diverse family of flowering plants, but it is currently facing unprecedented risks of extinction (Cribb *et al.* 2003; Swarts and Dixon 2009). Orchidaceae consists of over 1000 genera and most orchid genera contain one or more threatened or endangered species (Cribb *et al.* 2003). The majority of threatened orchid species are terrestrial orchids, despite the small portion of the family represented by this life form (IUCN 2001). In addition, many terrestrial orchids are rare, with specialized habitat requirements, making them particularly susceptible to habitat fragmentation and modification (Wu and Smeins 2000; Pillon and Chase 2007). Their vulnerability is exacerbated by patchy distributions, specialized mutualisms and generally limited dispersal (Schemske *et al.* 1994; Coates *et al.* 2006; Pillon and Chase 2007). Given the high extinction risk to terrestrial orchids, they have been a major conservation concern for many environmental groups. They have often been used as flagship species in conservation initiatives because of their uniqueness and rarity and additionally are often touted as important early warning bioindicators for ecosystem health given their sensitivity to environmental degradation (Cribb *et al.* 2003; Swarts and Dixon 2009).

Despite substantial conservation emphasis on rare orchids, populations continue to decline (Swarts and Dixon 2009). This is in no small part due to difficulties in designing integrated conservation plans for orchid protection (Whigham and Willems 2003; Swarts *et al.* 2007). Often, small patchily distributed orchid populations are difficult to detect without thorough surveys of extensive areas, which are often not logistically feasible (Wu and Smeins 2000; Gogol-Prokurat 2011). In addition, many populations are spread across a network of private or otherwise inaccessible land. Incomplete censuses of populations limit the ability for conservation planners to determine appropriate areas for protected habitat and assisted migrations (Cuperus *et al.* 1999; Wan *et al.* 2014). Effective conservation planning requires the identification of areas of suitable habitat in order to facilitate prioritization and appropriately identify land for the creation of preserves or easements and mitigation for habitat modification or loss (Rodríguez *et al.* 2007; Gogol-Prokurat 2011; Dudley and Bean 2012).

Species distribution models are invaluable tools for focussing conservation efforts of species with incomplete distribution records (Fleishman *et al.* 2002; Buse *et al.* 2007; Fandohan *et al.* 2011; Gogol-Prokurat 2011). Species distribution models comprise a suite of quantitative tools that statistically relate species presence and absence data to environmental predictor variables (Guisan and Thuiller 2005; Gogol-Prokurat 2011). They elucidate habitat requirements, aiding in the development of distribution predictions essential to meeting endangered species conservation objectives with limited site occupancy data and resources for additional data collection (Guisan and Thuiller 2005; Hirzel *et al.* 2006). They can be used to identify habitat suitable for conservation by providing maps of probabilities that the species would occur in a given area (Ibisch *et al.* 2002; Jiménez-Valverde and Lobo 2007), determine the effects of land-use change on endangered species habitat (Rodríguez *et al.* 2007) and explore the effect of global change on endangered species distributions (Jiménez-Valverde and Lobo 2007; Thuiller *et al.* 2008).

Studies involving species distribution modelling have increased in recent years and several methods are currently applied to address ecological issues (Elith and Leathwick 2009). These include statistical models such as generalized linear models (Wang and Grant 2012) and generalized additive models (Leathwick *et al.* 2006), machine-learning models such as CLIMEX (Pattison and Mack 2008), GARP (Stockman *et al.* 2006) and Maxent (Wilting *et al.* 2010), as well as methods drawing on insights and techniques from statistical and machine learning approaches such as random forests (Prasad *et al.* 2006) and boosted regression trees (Chiou *et al.* 2013). Boosted regression trees are a relatively new method compared to others. Boosted regression trees have their origins within machine learning, but subsequent developments in the statistical community have led to a reinterpretation of boosted regression trees as an advanced form of regression (Elith *et al.* 2008). However, boosted regression trees differ fundamentally from statistical methods and machine-learning approaches such as Maxent that produce a single ‘best’ model in that boosted regression trees combine a large number of relatively simple tree models adaptively to optimize predictive power (Leathwick *et al.* 2006). Each of the individual models consists of a simple classification or regression tree (a rule-based classifier) that partitions observations into groups having similar values for the response variable based on a series of binary rules constructed from the independent variables (Hastie *et al.* 2001). Boosted regression trees have been used to predict the distribution of a threatened species, rabbitsfoot (*Quadrula cylindrical*), via the inclusion of independent variables measured at markedly different spatial scales (Hopkins 2009).

In this study, we analysed the relationships between the occurrence of *Spiranthes parksii*, an endangered terrestrial orchid, and several climatic and landscape variables deemed important to *S. parksii* distribution. *Spiranthes parksii* is a state and federally listed endangered orchid, endemic to central Texas, USA. Its distribution is limited to 13 counties and it appears to occupy a restricted habitat within those counties, often observed on the edges of upland drainages in small open grass/shrub patches within post-oak savannah/woodland communities (Wonkka *et al.* 2012). *Spiranthes parksii* populations are threatened by land conversion to agriculture and lignite mines, urban development and woody encroachment by trees and understory shrubs into post-oak savannahs (Wonkka *et al.* 2012). Like many terrestrial orchids, *S. parksii* is a mycoheterotroph, requiring a mycorrhizal symbiont for germination and seedling development, remaining closely associated with the fungi throughout its life cycle (Ariza 2013). This specialized mutualism, along with a limited dispersal shadow, and specialized habitat requirements leads to a patchy distribution, making *S. parksii* detection difficult. In addition, the counties in which the populations are located consist largely of privately owned land, inaccessible to surveyors. High rates of development in this region necessitate effective prioritization of lands for mitigation efforts. Given the conservation concerns and limited availability of survey data for this species, we developed a species distribution model to aid in effective conservation of *S. parksii*. In particular, we used boosted regression trees to (i) identify potential factors influencing *S. parksii* distribution, (ii) quantify the relative importance of each factor and (iii) predict suitable *S. parksii* habitat. The model developed herein will provide an adaptive quantitative tool which can be used to facilitate future *S. parksii* surveying, research and conservation efforts and, with slight modification, should be applicable to other endangered species with similarly limited ranges.

## Methods

### Study area and data sources

**Figure 1.**
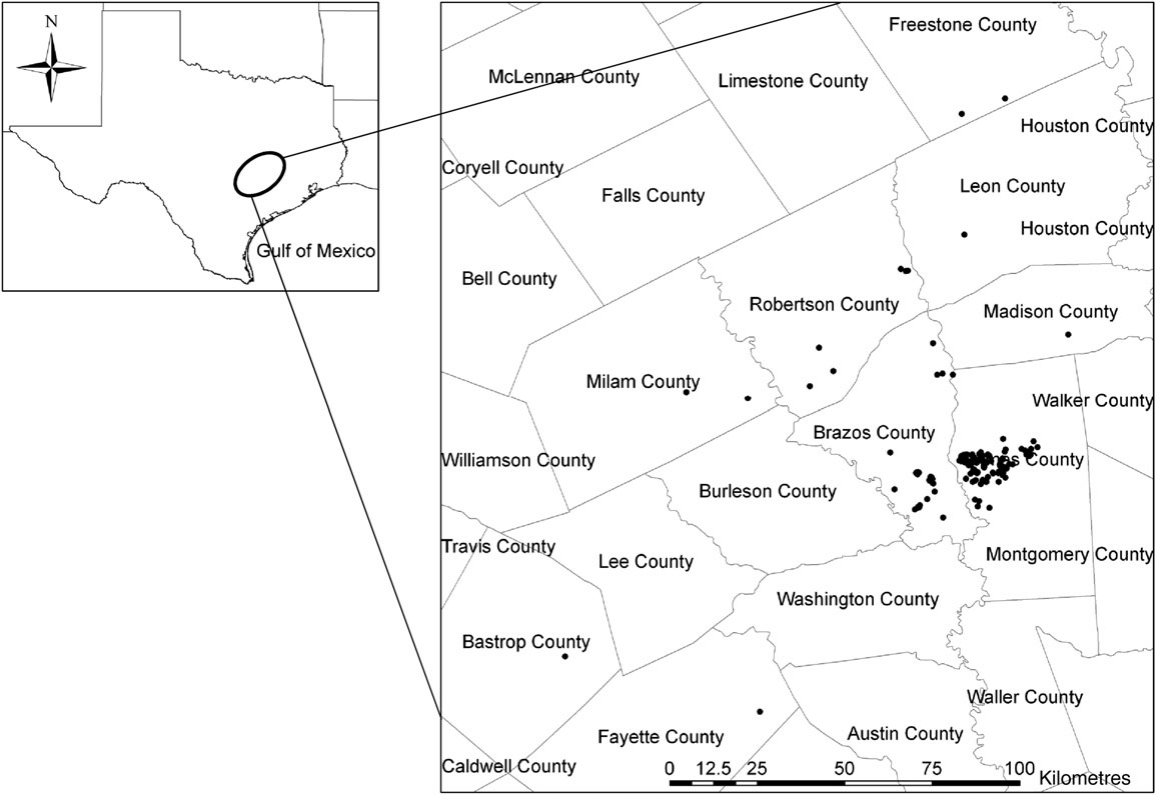
The study area and the current distribution (filled circles) of the endangered orchid *S. parksii* in central Texas, USA.

The study area covers several counties (Bastrop, Fayette, Milam, Freestone, Leon, Madison, Grimes, Robertson and Brazos) in central Texas, USA (Fig. 1). The area is largely post-oak savannah intermixed with open grassland, cropland and urban and suburban development. The climate is humid subtropical with an average minimum temperature of 14 °C, an average maximum temperature of 26 °C and average annual precipitation of 105 cm bimodally distributed with peaks in the fall and the spring.

We obtained geo-referenced data on (i) presence and absence of *S. parksii* regularly sampled between 2004 and 2012 from the US Fish and Wildlife Service, Texas Parks and Wildlife Department, Texas Department of Transportation and the Texas A&M University team working under Drs Smeins and Rogers, (ii) average climatic conditions in our study area from the PRISM Climate group (2013) and (iii) landscape features including topographic characteristics derived from digital elevation models (Gesch 2007), land cover (Fry *et al.* 2011), soil characteristics (Soil Data Mart 2013) and geology (Stoeser *et al.* 2013). *Spiranthes parksii* surveys were conducted yearly during peak flowering for the duration of the study with systematic sampling across areas of known *S. parksii* occupancy and additional areas deemed likely to contain *S. parksii* due to similarity in habitat characteristics to known areas of occupancy. *Spiranthes parksii* locations were marked with GPS coordinates for future observation.

Our geo-referenced data for *S. parksii* were primarily comprised of occurrences. Pseudo-absences, or random points in the study area where the focal species has not been documented, are typically used as a surrogate for absence records in studies with such data limitations. However, there may be limited confidence in absence at those points, depending on the sampling strategy (Phillips *et al.* 2009). As a more definitive sample of absence points, we used locality records for an ecologically similar congener, *S. cernua*. Given the similar ecology and phenology of the two species (Ariza 2013), and the conservation status of *S. parksii*, *S. parksii* would have been recorded if found during surveys for *S. cernua*. Thus, we confidently use records for *S. cernua* that lack concurrent records for *S. parksii*.

### Data analysis

We selected 58 variables that have been suggested in the literature as potential predictors of the presence of *S. parksii* in central Texas (Wonkka *et al.* 2012; Krupnick *et al.* 2013), including various climatic conditions and landscape features **[see Supporting Information]**. We analysed relationships between the occurrence of *S. parksii* and the potential explanatory variables by aggregating the explanatory variable data associated with *S. parksii* presence (106 cells) and absence (99 cells) into polygons representing a resolution of 800 × 800 m cells, aligned with the climate data that we used, in central Texas. We then merged these data into a grid of 37 427 800 × 800 m cells using ArcGIS 9.0 (ESRI 2009). The climate data included annual average maximum and minimum temperatures, and precipitation for 1981–2010 (PRISM Climate Group 2013). We derived topographic characteristics for the 800 m grid cells of analysis from the 30 m National Elevation Dataset (Gesch 2007) using SAGA GIS version 2.1.0 (www.sagagis.org). We also calculated the average soil water-holding capacity, percentage of silt, sand and clay in each soil type based on STATSGO soil data (Soil Data Mart 2013) using R version 3.0.2 (R Core Team 2013). We used spatial overlay tools in SAGA GIS version 2.1.0 and Manifold GIS version 8.0.28 to aggregate the various data layers **[see Supporting Information]** into a single dataset for analyses.

We conducted our analysis using boosted regression trees which combine decision trees and a boosting algorithm with a form of logistic regression (Friedman 2002; De'ath 2007; Elith *et al.* 2008). For boosted regression trees, the probability (*P*) of *S. parksii* occurrence (*y* = 1) at a location with the potential explanatory variables (*X*) is given by *P*(*y* = 1|*X*) and is modelled via the logit: logit *P*(*y* = 1|*X*) = *f*(*X*). We fitted our model in R (R Development Core Team 2006 version 2.14.1) using the gbm package version 1.5-7 (Ridgeway 2006) and code provided by Elith *et al.* (2008). The optimal model was determined following the recommendations of Elith *et al.* (2008) by altering the learning rate and tree complexity (the number of split nodes in a tree) until the predictive deviance was minimized without over-fitting, and by limiting our choice of the final model to those that contained at least 1000 trees (where each successive tree is built for the prediction residuals of the preceding tree). Once the optimal combination of learning rate and tree complexity was found, model performance was evaluated using a 10-fold cross-validation procedure with resubstitution. For each cross-validation trial, 80 % of the dataset was randomly selected for model fitting and the excluded 20 % was used for testing. We calculated the response variance explained, the area under the receiver operator characteristic curve (AUC), the overall accuracy, the omission error rate and the commission error rate based on the aggregated CV results. We evaluated the reliability and validity of our models as fair (0.50 < AUC ≤ 0.75), good (0.75 < AUC ≤ 0.92), very good (0.92 < AUC ≤ 0.97), or excellent (0.97 < AUC ≤ 1.00) based on the value of AUC (Hosmer and Lemeshow 2000). We then used the gbm library to derive the relative influence of each potential explanatory variable in the model and constructed partial dependence plots for the most influential variables (Elith *et al.* 2008). Finally, we used this optimal model to calculate probability of *S. parksii* presence in each cell in central Texas and superimposed these probabilities of occupancy on a map of the study area using ArcMap 9.0 (ESRI 2009).

## Results

Analyses of 500 combinations of tree complexity (ranging from 3 to 7) and learning rate (ranging from 0.001 to 0.01) produced models with between 450 and 3900 trees whose values of predictive deviance ranged from 0.582 to 0.624. The optimal model had a tree complexity of 5, a learning rate of 0.003 and a total of 1200 trees. Model predictive deviance was 0.582 ± 0.0079 with 95.6 % of the total response variance explained. The AUC score was 0.940 ± 0.016 (‘very good’ ability to discriminate between species presence and absence) and the overall accuracy was 91.7 %. The commission (false positive) error rate was 6.8 % and the omission (false negative) error rate was 9.8 %. Recursive feature elimination tests showed that 45 variables could be removed from the model before the resulting predictive deviance exceeded the initial predictive deviance of the model with all variables.

**Figure 2.**
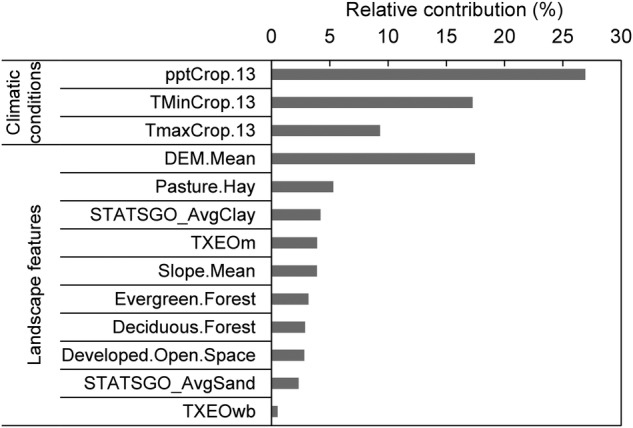
Relative contributions (%) of the 13 most influential variables included in the final model (see Table 1 for the description of variables).

Thirteen variables were included in the final model (Table 1), with variables associated with climatic conditions and landscape features accounting for ∼53.5 and 46.5 %, respectively, of the contribution in the overall model (Fig. 2). Examination of the relative contribution of the predictor variables indicated that the top four accounted for ∼70.95 % of the contribution in the overall model. Of the four most influential model variables, three were climatic conditions and one was a landscape feature. Mean annual precipitation, mean annual minimum temperature and mean annual maximum temperature were the first, third and fourth most influential variables, contributing 26.93, 17.26 and 9.31 %, respectively. Mean elevation was the second most important variable contributing 17.45 %.

**Table 1.** Abbreviations, descriptions and descriptive statistics for the climatic conditions and landscape features included in the final model.

Variable	Description	Mean	Minimum	Maximum
Climatic conditions				
pptCrop.13	Mean annual precipitation (mm × 100)	104 742	88 153	115 293
TMinCrop.13	Mean annual minimum temperature (C × 100)	1372	1255	1438
	Mean annual maximum temperature (C × 100)	2598	2499	2671
Landscape features				
DEM.Mean	Mean elevation (m)	101.49	48.40	194.61
Pasture.Hay	Proportion of pasture (%)	0.36	0	0.97
STATSGO_AvgClay	Percentage of clay based on average of soil types (%)	28.27	5.04	51.91
TXEOm	Percentage of Manning formation on average of geological formation (%)	0.29	0	1
Slope.Mean	Mean slope (degree × 100)	0.03	0	0.09
Evergreen.Forest	Proportion of evergreen forest (%)	0.05	0	0.73
Deciduous.Forest	Proportion of deciduous forest (%)	0.14	0	0.73
Developed.Open.Space	Percentage of developed open space (%)	0.04	0	0.61
STATSGO_AvgSand	Percentage of sand based on average of soil types (%)	42.57	15.53	94.30
TXEOwb	Percentage of Wellborn formation on average of geological formation (%)	0.06	0	1

**Figure 3.**
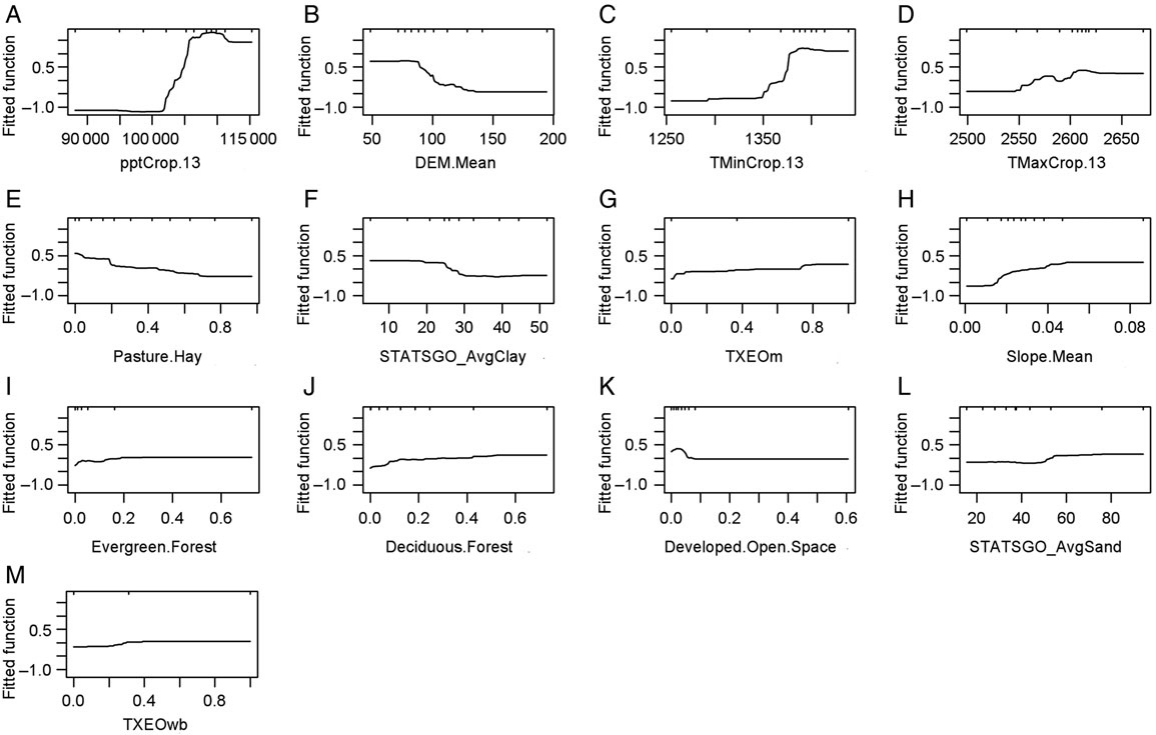
Partial dependence plots for the 13 most influential variables included in the final model. The *y*-axis represents the logit scale used for the indicated variable, hash marks at the top of the plot indicate the locations of the sample sites along the range of the variables.

Partial dependence plots indicated that *S. parksii* occurrences were associated with climatic conditions characterized by mean annual precipitation between 1050 and 1120 mm (Fig. 3A), mean annual minimum temperature between 13.75 and 14.38 °C (Fig. 3C) and mean annual maximum temperature between 26.00 and 26.25 °C (Fig. 3D). Occurrences also were associated with landscape features characterized by (i) an altitude between 50 and 80 m (Fig. 3B), (ii) a slope ratio between 0.05 and 0.09 % (Fig. 3H), (iii) areas with <20 % pasture (Fig. 3E), 20–73 % of evergreen forest (Fig. 3I), 50–73 % of deciduous forest (Fig. 3J), or <5 % of developed open space (Fig. 3K), (iv) soil with <20 % clay (Fig. 3F) or 55–94 % sand (Fig. 3L) and (v) geological formations in which >75 % belonged to the Manning formation (Fig. 3G) or in which >40 % belonged to the Wellborn formation (Fig. 3M).

**Figure 4.**
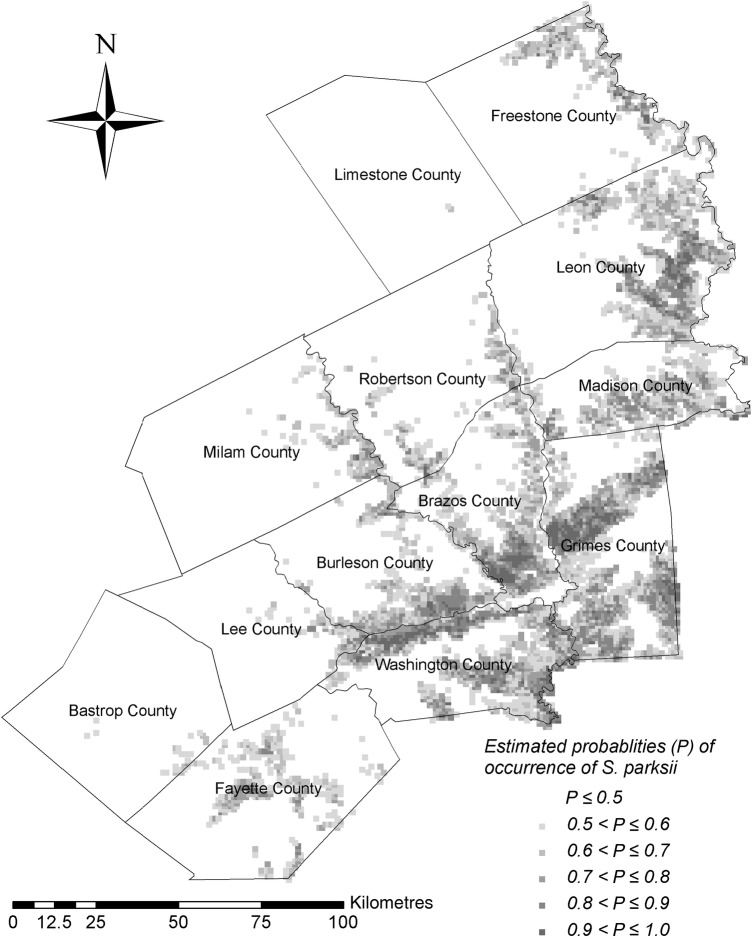
Estimated probabilities of occurrence of *S. parksii* in central Texas, USA.

Our analyses suggest that potential habitat for *S. parksii* in central Texas, considering its association with the variables mentioned in the previous paragraph, is most likely to be (i) the eastern portions of Leon and Madison Counties, (ii) the southern portion of Brazos County, (iii) a portion of northern Grimes County and (iv) along the borders between Burleson and Washington Counties (Fig. 4). Approximately 84, 5, 4, 3, 3 and 1 % of the cells fell within the *P* ≤ 0.5, 0.5 < *P* ≤ 0.6, 0.6 < *P* ≤ 0.7, 0.7 < *P* ≤ 0.8, 0.8 < *P* ≤ 0.9 and 0.9 < *P* ≤ 1.0 estimated probability of occurrence (*P*) categories, respectively.

## Discussion

Plant distributions are limited by the availability of suitable habitats (Aitken *et al*. 2007). For rare plants, especially those with limited geographic ranges, narrow habitat specificity can further limit distribution. While climate is an important determinant of plant distribution at landscape levels (Pearson *et al*. 2004), soil properties and biotic interactions determine habitat availability at local scales (Raven 2002). Even for edaphic endemics, combinations of variables predict distributions more accurately than simple models driven entirely by soil-related parameters (Arundel 2005). This is especially valid for predicting orchid species distributions which are highly dependent on interactions with pollinators and mycorrhizal symbionts (Rasmussen 2002; Rasmussen and Rasmussen 2009).

Our model establishes the importance of both climatic variables and landscape features to the distribution of *S. parksii*. *Spiranthes parksii* is associated with the higher end of the range of average annual precipitation for the area (1050–1120 mm). This agrees with findings by Ariza (2013) that showed higher soil moisture as a major explanatory variable differentiating *S. parksii* occurrence with the more abundant sympatric species *S. cernua*. *Spiranthes parksii* is also found in areas with high minimum (13.8–14.4 °C) and maximum (26–26.3 °C) mean annual temperatures. This likely contributes to *S. parksii* distribution through unique life-history characteristics including summer dormancy and potential early fall emergence of rosettes (Wonkka *et al*. 2012). Summer dormant plants existing below the soil as rhizomes can withstand high peak temperatures, but with above-ground photosynthetic vegetation being present in the winter, the plants favour areas with higher winter temperatures to minimize potential frost damage.

Given the importance of climatic variables to *S. parksii* distribution, climate change could have an extensive impact on the availability of suitable *S. parksii* habitat in the future. Climate change has been shown to cause distribution shifts for many species of plants and can increase the likelihood of local extinction as sessile plant species are unable to disperse or adapt to a rapidly changing climate (Hoegh-Guldberg *et al.* 2008; Nicolè *et al*. 2011; Parmesan *et al.* 2013). This is further exacerbated in highly fragmented areas, such as the range of *S. parksii*, where human alteration can act as a barrier to dispersal processes (Preston *et al.* 2008). Although there is considerable uncertainty in predictions of future temperatures and precipitation for a particular region, projections averaged across ensemble models suggest increased summer and winter temperatures and decreased average annual precipitation across the southern United States (Deser *et al.* 2012). In addition, precipitation is expected to become more variable, with more frequent drought events and more precipitation occurring in fewer rainfall events (Coumou and Rahmstorf 2012; Deser *et al.* 2012; Intergovernmental Panel on Climate Change 2014). While warmer temperatures could increase the habitat available to *S. parksii*, there is likely an upper bound on temperatures that the orchid can withstand. In addition, reduced precipitation and greater frequency of drought could cause many currently suitable areas of habitat to become too dry to support populations of the orchid given that our model shows *S. parksii* to occur in the wetter portions of its range.

Several landscape features also proved important to *S. parksii* distribution. Elevation and slope are also important to determining *S. parksii* occurrence. Although slope and elevation are not mechanistic variables, they often can be proxies for environmental variables, such as soil properties and plant-available water, which can drive plant distributions (Lassueur *et al*. 2006). Elevation, derived from digital elevation models, is the most important landscape feature for predicting *S. parksii* distribution. They are found at the low end of the elevation range for the area (50–80 m). This is reflective of the specific habitat preference for margins of drainages (Ariza 2013). Similarly, *S. parksii* occur in areas with maximum slope ratios for the area (0.05–0.09), which also reflects their occurrence between flatter open areas and margins of drainages.

Our model also showed soils and vegetation cover type to be important for the distribution of *S. parksii*. This is consistent with past studies that found high *S. parksii* occurrence in the Manning and Wellborn geological formations, suggesting that *S. parksii* might be an edaphic endemic. This is also consistent with the life cycle dependence on mycorrhizal fungi (Ariza 2013). Orchid distributions are thought to be restricted largely by interactions with pollinators and their mycorrhizal symbionts (Waterman and Bidartondo 2008). For *S. parksii*, pollination is likely less important (as evidenced by high levels of asexual reproduction) than fungal mutualism (Ariza 2013). Fungi tend to be patchily distributed across a landscape (Batty *et al*. 2001), and their distributions are driven largely by local mechanisms, especially soil properties such as soil moisture and soil organic matter (Brundrett and Abbott 1994). Ariza (2013) found higher summer soil moisture, lower pH, percent sand and abundance of soil organic matter to be the most important distinguishing characteristics between *S. parksii* and *S. cernua* occurrence. Our model suggests that *S. parksii* soils usually are found in areas with <20 % clay and 55–94 % sand. The importance of soil organic matter to *S. parksii* likely is related to both the ability of organic matter to increase the water-holding capacity of well-drained sandy soils, and also its importance as a substrate for fungi. Orchids also tend to exhibit narrow specificity with fungi (Waterman and Bidartondo 2008; Rasmussen and Rasmussen 2009). Therefore, it is likely that *S. parksii* distribution closely tracks particular fungal species. The distribution of those fungi is likely driven by specific soil inputs as well as soil properties, which could explain the importance of vegetative cover (<20 % pasture, 20–73 % evergreen, 50–73 % deciduous) to *S. parksii* distribution. Fungi likely require leaf litter as a substrate for decomposition. However, a thick layer of leaf litter might inhibit germination of *S. parksii* seeds. This is supported by the findings of Ariza (2013) that uniform leaf litter cover was an important determinant of *S. parksii* occurrence. Soils have been found to be important determinants of distribution for other orchids. Bowles *et al*. (2005) found soil type to be the most important variable determining *Plantanthera leucophaea* distribution and Clark *et al*. (2004) determined climate, soils and vegetation type to accurately predict distributions of *Cryptostylis hunteriana*.

Areas with high estimated probabilities of *S. parksii* occurrence are distributed patchily across the range. There are some larger connected areas of high probability, but many areas with high likelihood of occurrence are punctuated with lower likelihood patches. Plants with limited dispersal shadows are highly susceptible to local extinctions due to stochastic events in fragmented habitats (Bourg *et al*. 2005). The distribution map generated with our model suggests fragmented habitat for *S. parksii*, which has limited dispersal due to tiny seeds and the necessity for mycorrhizal associations for germination. Many seedlings are found in close proximity to adult plants (Ariza 2013). Patchy distributions often are associated with limited habitat availability (Swarts and Dixon 2009). However, if there is little chance for long distance dispersal and patchily distributed areas of suitable habitat (Hurtt and Pacala 1995), movement into unoccupied areas of suitable habitat could be restricted, posing problems for species with high frequencies of local extinctions (Primack and Miao 1992). This is especially significant in areas that are highly fragmented by development and agriculture such as the central Texas range of *S. parksii*. Additionally, distribution shifts necessary for species continuation in the face of climate change require areas of suitable habitat be attainable for future recruitment and persistence (Carey and Brown 1994). Existence of such sites becomes increasingly less likely with high habitat specificity, limited dispersal distances and fragmented habitat. Our model possesses potential utility for understanding *S. parksii* meta-population dynamics more thoroughly to determine the level of threat posed to species viability in the face of increased landscape fragmentation.

The model developed in this study has potential utility beyond the scope of this work. It can be adapted to incorporate new information and data as they become available. Model accuracy increases with increased amount and accuracy of presence and absence data and can be updated to include new information to further refine distribution predictions (Elith and Leathwick 2009). Additionally, modelling multiple scales could increase the accuracy of prediction. Ecological processes function at different scales (Turner 1989; Levin 1992). Our model explores landscape level scales that drive distribution, but refining the model resolution could yield important information regarding distribution at a finer scale (i.e. within a high probability patch). Fine scale mechanisms often regulate the distribution of rare plants with specialized habitat requirements (Menges *et al*. 1999). For instance, Diez and Pulliam (2007) found that distance from parent was important to germination at the local scale, while soil characteristics were more predictive of germination at larger scales for *Goodyera pubescencs*. One opportunity for improvement of the model is to incorporate data related to disturbance and biotic interactions (e.g. distribution of pollinators or fungal associates, fire or flooding effects on habitat quality) in order to reflect the potential for non-equilibrium system functioning (Schröder and Seppelt 2006). This could prove especially important for species such as rare orchids that have specific biotic interactions (Ettema and Wardle 2002) and tend to respond to specific disturbance regimes (Clark *et al*. 2004). Models incorporating landscape changes and mechanistic drivers can better capture fluctuations in habitat suitability over time (Kearney *et al*. 2008). This could increase the accuracy of distribution predictions in systems where habitat quality fluctuates in response to non-equilibrium processes (Elith and Leathwick 2009).

## Conclusions

A suite of climatic variables and landscape features can be used to predict the distribution of the endangered terrestrial orchid, *S. parksii* which is endemic to central Texas. Many of these variables are related to soil resources which potentially influence the distribution of the mycorrhizal fungi the orchid depends on for germination and lifetime nutrient acquisition (Rasmussen and Rasmussen 2007; Ariza 2013). The species' potential habitat is patchily distributed as a result of this dependence on soil resources and specific habitat requirements (Batty *et al*. 2001). Narrow habitat specificity combined with potential dispersal limitations necessitates an integrated conservation approach that includes research to determine basic ecological and biological processes important to *S. parksii* population viability, habitat management and conservation and an understanding of the effects of fragmentation and habitat degradation on dispersal of *S. parksii* into suitable habitat (Swarts and Dixon 2009). Species distribution models can assist in the development of an integrated conservation strategy (Kiesecker *et al*. 2010). They can help to fill knowledge gaps resulting from limited resources for research. Similarly, they can help focus future survey and research efforts on areas with a high likelihood of occurrence (Parris 2002; Guisan *et al*. 2006). Species distribution models also can be used to select areas for conservation offsets or easements (Gibbons and Lindenmayer 2007; Kumar and Stohlgren 2009), explore alternate management scenarios (Guisan *et al*. 2013), frame research questions (Aitken *et al*. 2007), explore issues related to meta-population dynamics (Bourg *et al*. 2005) and predict potential responses to climate change (Carey and Brown 1994). The species distribution model developed through this research is adaptive. It can incorporate new information as it becomes available to improve accuracy and resolution of our analyses. Our methodology could also be employed to develop distribution maps for other rare species of conservation concern.

## Sources of Funding

This research was funded by the City of Bryan/College Station-Brazos Valley Solid Waste Management Agency, Texas Department of Transportation and a Ladybird Johnson Wildflower Center (Austin, TX)—Endangered Species Conservation Grant Program Award #12419. C.L.W. was supported by a USDA-CSREES National Needs Fellowship (2009-38420-05631) and M.L.T. was supported by an NSF-IGERT Traineeship through the Applied Biodiversity Science Program at Texas A&M University (NSF DGE 0654377). C.L.W. and M.L.T. were also supported by the Tom Slick Doctoral Fellowship Program.

## Contributions by the Authors

All authors shared in collecting data, constructing the model and writing.

## Conflict of Interest Statement

None declared.

## Supporting Information

The following additional information is available in the online version of this article –

**Appendix.** Abbreviations, descriptions and descriptive statistics for the 94 climatic conditions and landscape features identified as potential factors influencing the likelihood of existence of *S. parksii* in the central Texas, USA.

Additional Information

## References

[PLV039C1] Aitken M Roberts DW Shultz LM 2007 Modeling distributions of rare plants in the Great Basin, western North America. Western North American Naturalist 67:26–38. 10.3398/1527-0904(2007)67[26:MDORPI]2.0.CO;2

[PLV039C2] Ariza MC 2013 Mycorrhizal associations, life history, and habitat characteristics of the endangered terrestrial orchid Spiranthes parksii corell and sympatric congener Spiranthes cernua: implications for conservation. PhD dissertation, Texas A&M University, College Station, TX.

[PLV039C3] Arundel ST 2005 Using spatial models to establish climatic limiters of plant species’ distributions. Ecological Modelling 182:159–181. 10.1016/j.ecolmodel.2004.07.016

[PLV039C5] Batty AL Dixon KW Brundrett M Sivasithamparam K 2001 Constraints to symbiotic germination of terrestrial orchid seed in a mediterranean bushland. New Phytologist 152:511–520. 10.1046/j.0028-646X.2001.00277.x 33862990

[PLV039C6] Bourg NA McShea WJ Gill DE 2005 Putting a CART before the search: successful habitat prediction for a rare forest herb. Ecology 86:2793–2804. 10.1890/04-1666

[PLV039C7] Bowles M Zettler L Bell T Kelsey P 2005 Relationships between soil characteristics, distribution and restoration potential of the federal threatened eastern prairie fringed orchid, *Platanthera leucophaea* (Nutt.) Lindl. The American Midland Naturalist 154:273–285. 10.1674/0003-0031(2005)154[0273:RBSCDA]2.0.CO;2

[PLV039C8] Brigham CA Swartz MA (eds) 2003 Population viability in plants: conservation, management, and modeling of rare plants. Berlin: Springer.

[PLV039C9] Brundrett MC Abbott LK 1994 Mycorrhizal fungus propagules in the Jarrah forest. I. Seasonal study of inoculum levels. New Phytologist 127:539–546. 10.1111/j.1469-8137.1994.tb03972.x 33863116

[PLV039C10] Buse J Schröder B Assmann T 2007 Modelling habitat and spatial distribution of an endangered longhorn beetle—A case study for saproxylic insect conservation. Biological Conservation 137:372–381. 10.1016/j.biocon.2007.02.025

[PLV039C11] Carey PD Brown NJ 1994 The use of GIS to identify sites that will become suitable for a rare orchid, *Himantoglossum hircinum* L., in a future changed climate. Biodiversity Letters 2:117–123. 10.2307/2999715

[PLV039C13] Chiou C-R Wang H-H Chen Y-J Grant WE Lu M-L 2013 Modeling potential range expansion of the invasive shrub *Leucaena leucocephala* in the Hengchun peninsula, Taiwan. Invasive Plant Science and Management 6:492–501. 10.1614/IPSM-D-13-00010.1

[PLV039C14] Clark S deLacey C Chamberlain S 2004 Using environmental variables and multivariate analysis to delineate preferred habitat for *Cryptostylis hunteriana*, the leafless tongue orchid, in the Shoalhaven local government area, NSW. Cunninghamia 8:467–476.

[PLV039C15] Coates F Lunt ID Tremblay RL 2006 Effects of disturbance on population dynamics of the threatened orchid *Prasophyllum correctum* D.L. Jones and implications for grassland management in south-eastern Australia. Biological Conservation 129:59–69. 10.1016/j.biocon.2005.06.037

[PLV039C16] Coumou D Rahmstorf S 2012 A decade of weather extremes. Nature Climate Change 2:491–496.

[PLV039C17] Cribb PJ Kell SP Dixon KW Barrett RL 2003 Orchid conservation: a global perspective. In: Dixon KW Kell SP Barrett RL Cribb PJ, eds. Orchid conservation. Kota Kinabalu, Sabah: Natural History Publications, 1–24.

[PLV039C18] Cuperus R Canters KJ Udo de Haes HA Friedman DS 1999 Guidelines for ecological compensation associated with highways. Biological Conservation 90:41–51. 10.1016/S0006-3207(99)00007-5

[PLV039C19] De'ath G 2007 Boosted trees for ecological modeling and prediction. Ecology 88:243–251. 10.1890/0012-9658(2007)88[243:BTFEMA]2.0.CO;2 17489472

[PLV039C20] Deser C Knutti R Solomon S Phillips AS 2012 Communication of the role of natural variability in future North American climate. Nature Climate Change 2:775–779. 10.1038/nclimate1562

[PLV039C21] Diez JM Pulliam HR 2007 Hierarchical analysis of species distributions and abundance across environmental gradients. Ecology 88:3144–3152. 10.1890/07-0047.1 18229848

[PLV039C22] Dudley TL Bean DW 2012 Tamarisk biocontrol, endangered species risk and resolution of conflict through riparian restoration. BioControl 57:331–347. 10.1007/s10526-011-9436-9

[PLV039C23] Elith J Leathwick JR 2009 Species distribution models: ecological explanation and prediction across space and time. Annual Review of Ecology, Evolution, and Systematics 40:677–697. 10.1146/annurev.ecolsys.110308.120159

[PLV039C24] Elith J Leathwick JR Hastie T 2008 A working guide to boosted regression trees. Journal of Animal Ecology 77:802–813. 10.1111/j.1365-2656.2008.01390.x 18397250

[PLV039C25] ESRI. 2009 ArcGIS. Redlands, CA: Environmental Systems Research Institute.

[PLV039C26] Ettema CH Wardle DA 2002 Spatial soil ecology. Trends in Ecology and Evolution 17:177–183. 10.1016/S0169-5347(02)02496-5

[PLV039C27] Falk DA Holsinger KE (eds) 1991 Genetics and conservation of rare plants. New York: Oxford University Press.

[PLV039C28] Fandohan B Assogbadjo AE Glèlè Kakaï RL Sinsin B 2011 Effectiveness of a protected areas network in the conservation of *Tamarindus indica* (Leguminosea-Caesalpinioideae) in Benin. African Journal of Ecology 49:40–50. 10.1111/j.1365-2028.2010.01228.x

[PLV039C29] Fleishman E Ray C Sjögren-Gulve P Boggs CL Murphy DD 2002 Assessing the roles of patch quality, area, and isolation in predicting metapopulation dynamics. Conservation Biology 16:706–716. 10.1046/j.1523-1739.2002.00539.x

[PLV039C30] Friedman JH 2002 Stochastic gradient boosting. Computational Statistics and Data Analysis 38:367–378. 10.1016/S0167-9473(01)00065-2

[PLV039C31] Fry JG Xian SJ Dewitz J Homer C Yang L Barnes C Herold N Wickham J 2011 Completion of the 2006 national land cover database for the conterminous United States. Photogrammetric Engineering and Remote Sensing 77:858–864.

[PLV039C32] Gesch DB 2007 The national elevation dataset. In: Maune D, ed. Digital elevation model technologies and applications: the DEM user's manual, 2nd edn Bethesda, MD: American Society for Photogrammetry and Remote Sensing, 99–118.

[PLV039C33] Gibbons P Lindenmayer DB 2007 Offsets for land clearing: no net loss or the tail wagging the dog? Ecological Management and Restoration 8:26–31. 10.1111/j.1442-8903.2007.00328.x

[PLV039C34] Gogol-Prokurat M 2011 Predicting habitat suitability for rare plants at local spatial scales using a species distribution model. Ecological Applications 21:33–47. 10.1890/09-1190.1 21516886

[PLV039C35] Guisan A Thuiller W 2005 Predicting species distribution: offering more than simple habitat models. Ecology Letters 8:993–1009. 10.1111/j.1461-0248.2005.00792.x 34517687

[PLV039C36] Guisan A Broennimann O Engler R Vust M Yoccoz NG Lehmann A Zimmermann NE 2006 Using niche-based models to improve the sampling of rare species. Conservation Biology 20:501–511. 10.1111/j.1523-1739.2006.00354.x 16903111

[PLV039C37] Guisan A Tingley R Baumgartner JB Naujokaitis-Lewis I Sutcliffe PR Tulloch AIT Regan TJ Brotons L McDonald-Madden E Mantyka-Pringle C Martin TG Rhodes JR Maggini R Setterfield SA Elith J Schwartz MW Wintle BA Broennimann O Austin M Ferrier S Kearney MR Possingham HP Buckley YM 2013 Predicting species distributions for conservation decisions. Ecology Letters 16:1424–1435. 10.1111/ele.12189 24134332 PMC4280402

[PLV039C38] Hastie T Tibshirani R Friedman JH 2001 The elements of statistical learning: data mining, inference, and prediction. New York, NY: Springer.

[PLV039C39] Hirzel AH Le Lay G Helfer V Randin C Guisan A 2006 Evaluating the ability of habitat suitability models to predict species presences. Ecological Modelling 199:142–152. 10.1016/j.ecolmodel.2006.05.017

[PLV039C40] Hoegh-Guldberg O Hughes L McIntyre S Lindenmayer DB Parmesan C Possingham HP Thomas CD 2008 Assisted colonization and rapid climate change. Science 321:345–346. 10.1126/science.1157897 18635780

[PLV039C41] Hopkins RL 2009 Use of landscape pattern metrics and multiscale data in aquatic species distribution models: a case study of a freshwater mussel. Landscape Ecology 24:943–955. 10.1007/s10980-009-9373-5

[PLV039C42] Hosmer DW Lemeshow S 2000 Applied logistic regression. New York, NY: John Wiley and Sons, Inc.

[PLV039C43] Hurtt GC Pacala SW 1995 The consequences of recruitment limitation: reconciling chance, history and competitive differences between plants. Journal of Theoretical Biology 176:1–12. 10.1006/jtbi.1995.0170

[PLV039C44] Ibisch PL Nowicki C Müller R Araujo N 2002 Methods for the assessment of habitat and species conservation status in data-poor countries–case study of the Pleurothallidinae (Orchidaceae) of the Andean rain forests of Bolivia. In: Bussman RW Lange S, eds. Conservation of biodiversity in the Andes and the Amazon. Proceedings of the Andes and the Amazon Basin Conference, Cusco, Peru. INKA e.V., Munich, Germany, 225–246.

[PLV039C45] Intergovernmental Panel on Climate Change. 2014 Climate change 2013: the physical science basis: working group I contribution to the fifth assessment report of the intergovernmental panel on climate change. Cambridge: Cambridge University Press.

[PLV039C46] IUCN. 2001 IUCN Red List categories and criteria: version 3.1. Prepared by the IUCN Species Survival Commission.

[PLV039C47] Jiménez-Valverde A Lobo JM 2007 Threshold criteria for conversion of probability of species presence to either—or presence—absence. Acta Oecologica 31:361–369. 10.1016/j.actao.2007.02.001

[PLV039C48] Kearney M Phillips BL Tracy CR Christian KA Betts G Porter WP 2008 Modelling species distributions without using species distributions: the cane toad in Australia under current and future climates. Ecography 31:423–434. 10.1111/j.0906-7590.2008.05457.x

[PLV039C49] Kiesecker JM Copeland H Pocewicz A McKenney B 2010 Development by design: blending landscape-level planning with the mitigation hierarchy. Frontiers in Ecology and the Environment 8:261–266. 10.1890/090005

[PLV039C50] Krupnick GA McCormick MK Mirenda T Whigham DF 2013 The status and future of orchid conservation in north America. Annals of the Missouri Botanical Garden 99:180–198. 10.3417/2011108

[PLV039C51] Kumar S Stohlgren TJ 2009 Maxent modeling for predicting suitable habitat for threatened and endangered tree *Canacomyrica monticola* in New Caledonia. Journal of Ecology and the Natural Environment 1:94–98.

[PLV039C52] Lassueur T Joost SP Randin CF 2006 Very high resolution digital elevation models: do they improve models of plant species distribution? Ecological Modelling 198:139–153. 10.1016/j.ecolmodel.2006.04.004

[PLV039C53] Leathwick JR Elith J Hastie T 2006 Comparative performance of generalized additive models and multivariate adaptive regression splines for statistical modelling of species distributions. Ecological Modelling 199:188–196. 10.1016/j.ecolmodel.2006.05.022

[PLV039C54] Levin SA 1992 The problem of pattern and scale in ecology: the Robert H. MacArthur Award Lecture. Ecology 73:1943–1967. 10.2307/1941447

[PLV039C55] Maschinski J Haskins KE (eds) 2012 Plant reintroduction in a changing climate: promises and perils. Washington, DC: Island Press.

[PLV039C56] Menges ES McIntyre PJ Finer MS Goss E Yahr R 1999 Microhabitat of the narrow Florida scrub endemic *Dicerandra christmanii*, with comparisons to its congener *D. frutescens*. Journal of the Torrey Botanical Society 126:24–31. 10.2307/2997252

[PLV039C57] Nicolè F Dahlgren JP Vivat A Till-Bottraud I Ehrlén J 2011 Interdependent effects of habitat quality and climate on population growth of an endangered plant. Journal of Ecology 99:1211–1218. 10.1111/j.1365-2745.2011.01852.x

[PLV039C58] Parmesan C Burrows MT Duarte CM Poloczanska ES Richardson AJ Schoeman DS Singer MC 2013 Beyond climate change attribution in conservation and ecological research. Ecology Letters 16:58–71. 10.1111/ele.12098 23679010

[PLV039C59] Parris KM 2002 More bang for your buck: the effect of caller position, habitat and chorus noise on the efficiency of calling in the spring peeper. Ecological Modelling 156:213–224. 10.1016/S0304-3800(02)00170-9

[PLV039C60] Pattison RR Mack RN 2008 Potential distribution of the invasive tree *Triadica sebifera* (Euphorbiaceae) in the United States: evaluating climex predictions with field trials. Global Change Biology 14:813–826. 10.1111/j.1365-2486.2007.01528.x

[PLV039C61] Pearson RG Dawson TP Liu C 2004 Modelling species distributions in Britain: a hierarchical integration of climate and land-cover data. Ecography 27:285–298. 10.1111/j.0906-7590.2004.03740.x

[PLV039C62] Phillips SJ Dudík M Elith J Graham CH Lehmann A Leathwick J Ferrier S 2009 Sample selection bias and presence-only distribution models: implications for background and pseudo-absence data. Ecological Applications 19:181–197. 10.1890/07-2153.1 19323182

[PLV039C63] Pillon Y Chase MW 2007 Taxonomic exaggeration and its effects on orchid conservation. Conservation Biology 21:263–265. 10.1111/j.1523-1739.2006.00573.x 17298532

[PLV039C64] Pitman NCA Jorgensen PM 2002 Estimating the size of the world's threatened flora. Science 298:989 10.1126/science.298.5595.989 12411696

[PLV039C65] Prasad AM Iverson LR Liaw A 2006 Newer classification and regression tree techniques: bagging and random forests for ecological prediction. Ecosystems 9:181–199. 10.1007/s10021-005-0054-1

[PLV039C66] Preston KL Rotenberry JT Redak RA Allen MF 2008 Habitat shifts of endangered species under altered climate conditions: importance of biotic interactions. Global Change Biology 14:2501–2515.

[PLV039C67] Primack RB Miao SL 1992 Dispersal can limit local plant distribution. Conservation Biology 6:513–519. 10.1046/j.1523-1739.1992.06040513.x

[PLV039C68] PRISM Climate Group. 2013 Corvallis, OR: Oregon State University http://prism.oregonstate.edu.

[PLV039C69] Rasmussen HN 2002 Recent developments in the study of orchid mycorrhiza. Plant and Soil 244:149–163. 10.1023/A:1020246715436

[PLV039C70] Rasmussen HN Rasmussen FN 2007 Trophic relationships in orchid mycorrhiza-diversity and implications for conservation. Lankesteriana 7:334–341.

[PLV039C71] Rasmussen HN Rasmussen FN 2009 Orchid mycorrhiza: implications of a mycophagous life style. Oikos 118:334–345. 10.1111/j.1600-0706.2008.17116.x

[PLV039C72] Raven PH 2002 Predicting species occurrences: issues of accuracy and scale. Washington, DC.

[PLV039C73] R Core Team. 2013 R: a language and environment for statistical computing. Vienna, Austria: R Foundation for Statistical Computing http://www.R-project.org/.

[PLV039C74] Ridgeway G 2006 Generalized boosted regression models. Documentation on the R package ‘gbm’, version 1-5-7 http://www.i-pensieri.com/gregr/gbm.shtml.

[PLV039C75] Rodríguez JP Brotons L Bustamante J Seoane J 2007 The application of predictive modelling of species distribution to biodiversity conservation. Diversity and Distributions 13:243–251. 10.1111/j.1472-4642.2007.00356.x

[PLV039C76] Schemske DW Husband BC Ruckelshaus MH Goodwillie C Parker IM Bishop JG 1994 Evaluating approaches to the conservation of rare and endangered plants. Ecology 75:584–606. 10.2307/1941718

[PLV039C77] Schröder B Seppelt R 2006 Analysis of pattern–process interactions based on landscape models—Overview, general concepts, and methodological issues. Ecological Modelling 199:505–516. 10.1016/j.ecolmodel.2006.05.036

[PLV039C78] Soil Data Mart. 2013 U.S. General soil map (STATSGO2). USDA/NRCS http://soildatamart.nrcs.usda.gov.

[PLV039C79] Stockman AK Beamer DA Bond JE 2006 An evaluation of a GARP model as an approach to predicting the spatial distribution of non-vagile invertebrate species. Diversity and Distributions 12:81–89. 10.1111/j.1366-9516.2006.00225.x

[PLV039C80] Stoeser DB Shock N Green GN Dumonceaux GM Heran WD 2013 A digital geologic map database for the state of Texas: U.S. Geological Survey data series. Denver, CO: U.S. Geological Survey.

[PLV039C81] Swarts ND Dixon KW 2009 Terrestrial orchid conservation in the age of extinction. Annals of Botany 104:543–556. 10.1093/aob/mcp025 19218582 PMC2720663

[PLV039C82] Swarts ND Batty AL Hopper S Dixon K 2007 Does integrated conservation of terrestrial orchids work? Lankesteriana 7:219–222.

[PLV039C83] Thuiller W Albert C Araújo MB Berry PM Cabeza M Guisan A Hickler T Midgley GF Paterson J Schurr FM Sykes MT Zimmermann NE 2008 Predicting global change impacts on plant species’ distributions: future challenges. Perspectives in Plant Ecology, Evolution and Systematics 9:137–152. 10.1016/j.ppees.2007.09.004

[PLV039C84] Turner MG 1989 Landscape ecology: the effect of pattern on process. Annual Review of Ecology and Systematics 20:171–197. 10.1146/annurev.es.20.110189.001131

[PLV039C85] Wan J Wang C Han S Yu J 2014 Planning the priority protected areas of endangered orchid species in northeastern China. Biodiversity and Conservation 23:1395–1409. 10.1007/s10531-014-0671-0

[PLV039C86] Wang H-H Grant WE 2012 Determinants of Chinese and European privet (*Ligustrum sinense* and *Ligustrum vulgare*) invasion and likelihood of further invasion in southern U.S. forestlands. Invasive Plant Science and Management 5:454–463. 10.1614/IPSM-D-12-00038.1

[PLV039C87] Waterman RJ Bidartondo MI 2008 Deception above, deception below: linking pollination and mycorrhizal biology of orchids. Journal of Experimental Botany 59:1085–1096. 10.1093/jxb/erm366 18316318

[PLV039C88] Whigham DF Willems JH 2003 Demographic studies and life-history strategies of temperate terrestrial orchids as a basis for conservation. In: Dixon KW Kell SP Barrett RL Cribb PJ, eds. Orchid conservation. Kota Kinabalu, Borneo: Natural History Publications, 137–158.

[PLV039C89] Wilcove DS Rothstein D Dubow J Phillips A Losos E 1998 Quantifying threats to imperiled species in the United States. BioScience 48:607–615. 10.2307/1313420

[PLV039C90] Wilting A Cord A Hearn AJ Hesse D Mohamed A Traeholdt C Cheyne SM Sunarto S Jayasilan M-A Ross J Shapiro AC Sebastian A Dech S Breitenmoser C Sanderson J Duckworth JW Hofer H 2010 Modelling the species distribution of flat-headed cats (*Prionailurus planiceps*), an endangered south-east Asian small felid. PLoS ONE 5:e9612 10.1371/journal.pone.0009612 20305809 PMC2840020

[PLV039C91] Wonkka CL Rogers WE Smeins FE Hammons JR Ariza MC Haller SJ 2012 Biology, ecology, and conservation of Navasota ladies-tresses (*Spiranthes parksii* Correll): an endangered terrestrial orchid of Texas. Native Plants Journal 13:236–243.

[PLV039C92] Wu XB Smeins FE 2000 Multiple-scale habitat modeling approach for rare plant conservation. Landscape and Urban Planning 51:11–28. 10.1016/S0169-2046(00)00095-5

