# Supplementary material for: Species distribution modelling for conservation of an endangered endemic orchid
Source: AoB Plants. 2015 Apr 21;7:plv039. doi: 10.1093/aobpla/plv039 (PMC4463238; doi:10.1093/aobpla/plv039)
Supplement: Additional Information [file supp_7_plv039_index.html]

Species distribution modelling for conservation of an endangered endemic orchid — Additional Information 

# Species distribution modelling for conservation of an endangered endemic orchid

## Additional Information

Additional Information

**Files in this Data Supplement:**

- Additional Information - Docx file
