# Supplementary material for: Species distribution modelling for conservation of an endangered endemic orchid
Source: AoB Plants. 2015 Apr 21;7:plv039. doi: 10.1093/aobpla/plv039 (PMC4463238; doi:10.1093/aobpla/plv039)
Supplement: Additional Information [file supp_plv039_aobplants-14144-s01.docx]

Appendix

Abbreviations, descriptions, and descriptive statistics for the 94 climatic conditions and landscape features identified as potential factors influencing the likelihood of existence of *S. parksii* in the central Texas, USA.

Climatic conditions:

pptCrop.13: Mean annual precipitation (mm×100)

TMaxCrop.13: Mean annual maximum temperature (C×100).

TMinCrop.13: Mean annual minimum temperature (C×100)

Landscape features

Distance: Distance from the cell centroid to nearest stream (degrees).

DEM.Mean: Mean elevation (m).

Mean slope: Mean slope (%, degree×100).

Aspect: Slope aspect.

Open.Water: Percentage of open water (%).

Developed.Open.Space: Percentage of developed open space (%).

Developed.Low.Intensity: Percentage of developed low intensity area (%).

Developed.Medium.Intensity: Percentage of developed medium intensity area (%).

Developed.High.Intensity: Percentage of developed high intensity area (%).

Barren.Land: Percentage of barren land (%).

Deciduous.Forest: Percentage of deciduous forest (%).

Evergreen.Forest: Percentage of evergreen forest (%).

Mixed.Forest: Percentage of mixed forest (%).

Shrub.Scrub: Percentage of shrub land (%)

Grassland.Herbaceous: Percentage of grassland (%).

Pasture.Hay: Percentage of pasture (%).

Cultivated.Crops: Percentage of cultivated land (%).

Woody.Wetlands: Percentage of woody wetland (%).

Emergent.Herbaceous.Wetlands: Percentage of emergent herbaceous wetland (%).

STATSGO_AvgSand: Percentage of sand based on average of soil types (%).

STATSGO_AvgSilt: Percentage of silt based on average of soil types (%).

STATSGO_AvgClay: Percentage of clay based on average of soil types (%).

STATSGO_AvgWaterStor: Percentage of water storage based on average of soil types (%).

TXEOPNc: Percentage of Calvert Bluff formation on average of geological formation (%).

TXEOPNwi: Percentage of Wilcox group on average of geological formation (%).

TXEoc: Percentage of Carizzo sand on average of geological formation (%).

TXEOca: Percentage of Cadell formation on average of geological formation (%).

TXEOcm: Percentage of Cook mountain formation on average of geological formation (%).

TXEOm: Percentage of Manning formation on average of geological formation (%).

TXEOqc: Percentage of Queen city sand on average of geological formation (%).

TXEOr: Percentage of Reklaw formation on average of geological formation (%).

TXEOs: Percentage of Sparta sand on average of geological formation (%).

TXEOw: Percentage of Weches formation on average of geological formation (%).

TXEOwb: Percentage of Wellborn formation on average of geological formation (%).

TXEOy: Percentage of Yegua formation on average of geological formation (%).

TXKkc: Percentage of Kemp clay and Corsicana Marl formation on average of geological formation (%).

TXKke: Percentage of Kemp clay on average of geological formation (%).

TXKnb: Percentage of Navarro group and Marlbrook Marl formation on average of geological formation (%).

TXKns: Percentage of Nacatoch sand on average of geological formation (%).

TXKnt: Percentage of Navarro and Taylor groups on average of geological formation (%).

TXKpg: Percentage of Pecan Gap Chalk formation on average of geological formation (%).

TXKu: Percentage of upper Cretaceous rocks on average of geological formation (%).

TXKwc: Percentage of Wolfe city formation on average of geological formation (%).

TXMIf: Percentage of Fleming formation on average of geological formation (%).

TXMIo: Percentage of Oakville sandstone on average of geological formation (%).

TXOGEOw: Percentage of Whitsett formation on average of geological formation (%).

TXOGc: Percentage of Catahoula formation on average of geological formation (%).

TXPNh: Percentage of Hooper formation on average of geological formation (%).

TXPNk: Percentage of Kincaid formation on average of geological formation (%).

TXPNmi: Percentage of Midway group on average of geological formation (%).

TXPNs: Percentage of Simsboro formation on average of geological formation (%).

TXPOw: Percentage of Willis formation on average of geological formation (%).

TXPNw: Percentage of Wills point formation on average of geological formation (%).

TXQal: Percentage of Alluvium formation on average of geological formation (%).

TXQd: Percentage of Deweyville formation on average of geological formation (%).

TXQhg: Percentage of High gravel deposits on average of geological formation (%).

TXQl: Percentage of Lissie formation on average of geological formation (%).

TXQt: Percentage of Terrace deposits on average of geological formation (%).

TXQu: Percentage of Quaternary deposit on average of geological formation (%).
